# Editorial: Advances in the treatment of sexual precocity and infertility

**DOI:** 10.3389/fendo.2024.1516736

**Published:** 2024-11-25

**Authors:** Dimitrios T. Papadimitriou, George Mastorakos, Constantine A. Stratakis

**Affiliations:** ^1^ Neonatal - Pediatric - Adolescent Endocrinology, Department of Pediatrics, University General Hospital of Larisa, Faculty of Medicine, University of Thessaly, Larisa, Greece; ^2^ Endocrine Unit, Aretaieion University Hospital, School of Medicine, National and Kapodistrian University of Athens, Athens, Greece; ^3^ Eunice Kennedy Shriver National Institute of Child Health and Human Development (NIH), Bethesda, MD, United States; ^4^ Human Genetics & Precision Medicine & DIGENIA Laboratory, Institute of Molecular Biology and Biotechnology of the Foundation for Research and Technology Hellas (IMMB-FORTH), Heraklion, Crete, Greece

**Keywords:** infertility, precocious puberty, hypogonadism, aromatase inhibitors, *in vitro* fertilization, personalized medicine, polycystic ovary syndrome

## Introduction

Our topic “Advances in the Treatment of Sexual Precocity and Infertility” addresses critical advancements in pediatric-adolescent endocrinology, reproductive medicine, and gynecology. With genetic precision medicine becoming increasingly important (1), this collection of 27 articles explores the underlying mechanisms of sexual precocity, hypogonadism, infertility (2), and offers novel insights into therapeutic approaches, enhancing both clinical outcomes and quality of life for affected individuals. The research emphasizes innovations in reproductive treatments, personalized medicine, hormonal therapies involving aromatase inhibitors even in early maturing girls with a compromised growth potential (3), and management strategies for conditions like polycystic ovary syndrome (PCOS) (4), poor ovarian response, and hypogonadotropic hypogonadism.

## Infertility treatments and protocol optimization

Several studies in this Research Topic focus on improving *in vitro* fertilization (IVF) protocols to optimize pregnancy outcomes while maintaining cost-effectiveness. A multicenter randomized controlled trial comparing a modified GnRH antagonist protocol based on luteinizing hormone (LH) levels with the conventional GnRH antagonist protocol found that both had similar clinical efficacy. However, the modified protocol was more cost-effective, reducing overall financial burden during assisted reproductive technology (ART) cycles (Liu et al.). The exploration of ovulation promotion protocols in young IVF patients with low anti-Müllerian hormone (AMH) levels revealed that the GnRH antagonist protocol yielded better cumulative live birth rates (CLBR) than the progestin-primed ovarian stimulation (PPOS) regimen, particularly for patients with low but not very low AMH levels. For very low AMH levels, both protocols were comparable in CLBR outcomes, suggesting that age and AMH status should guide protocol selection (Li et al.). Further extending this focus on individualized protocols, a study evaluating different starting doses of recombinant human follicle-stimulating hormone (rhFSH) in patients with normal ovarian reserves found that a starting dose of 150 IU resulted in a similar pregnancy outcome compared to a 225 IU dose. The lower starting dose was also associated with shorter time to live birth and reduced cost, making it an attractive option for normal ovarian reserve patients (Jia et al.). Similarly, a real-world study on follitropin delta, an innovative rhFSH, confirmed its effectiveness in IVF protocols. The study highlighted its favorable pregnancy rates, minimized ovarian hyperstimulation syndrome (OHSS) risk, and reliable dosing patterns in routine clinical practice (Blockeel et al.). Letrozole cotreatment has also gained attention, especially in women with polycystic ovary syndrome (PCOS) and high body mass index (BMI), who often experience attenuated ovarian responses to IVF. This study found that letrozole combined with PPOS improved the follicular output rate (FORT), although the overall pregnancy outcomes did not differ significantly from those without letrozole (Liu et al.). The effects of glucocorticoids and androgens on ovarian follicle function were examined in a study exploring stress-induced diminished ovarian reserve. Findings indicated that excessive glucocorticoids impaired ovarian function, but androgens could improve early-stage follicle development through synergistic signaling with insulin-like growth factor 1 (IGF1) and follicle-stimulating hormone (FSH) (Gao et al.). Endometrial receptivity, a key factor in implantation success, was the subject of a study comparing outcomes of Day 7 blastocyst transfer to Day 5 and Day 6 transfers. The Day 7 group showed significantly lower live birth and pregnancy rates, while preimplantation genetic testing (PGT) was suggested as a strategy to enhance outcomes for Day 7 transfers (Liu et al.). A critical study on vanishing twin syndrome (VTS) identified intrauterine hematoma (IUH) as a risk factor for VTS in twin pregnancies following IVF. The presence of IUH was associated with increased risks of preterm birth, threatened abortion, and postpartum hemorrhage, emphasizing the need for close monitoring in twin pregnancies (Ge et al.).

## Hormonal ratios and IVF outcomes

Several studies explored hormonal markers as predictors of IVF outcomes. A study examining the follicle-stimulating hormone-to-luteinizing hormone (FSH/LH) ratio in women undergoing GnRH antagonist protocols found that this ratio could predict poor ovarian response and reproductive potential. The basal FSH/LH ratio was the strongest predictor, particularly for identifying poor responders and women with limited embryo availability (Zhao et al.). The progesterone-to-retrieved oocyte (P/O) ratio during the late follicular phase also emerged as a significant predictor of pregnancy outcomes in fresh embryo transfer cycles. Higher P/O ratios were associated with reduced live birth rates, suggesting that this marker can guide decision-making on embryo transfer strategies (Zhang et al.).

## Male infertility and advanced age

The role of the aging male in sperm quality and reproductive outcomes was addressed through a study assessing sperm chromatin integrity and hormonal markers in subfertile men. Older men (>40 years) exhibited increased sperm chromatin damage and chromatin immaturity compared to younger men, underscoring the impact of age on male fertility. These findings emphasize the importance of evaluating sperm integrity in older males undergoing ART (Bibi et al.).

## Traditional and complementary approaches

The Research Topic also highlights alternative therapies. A prospective study on acupuncture examined its effect on endometrial receptivity and implantation success in rats undergoing controlled ovarian hyperstimulation (COH). Acupuncture improved pregnancy rates by restoring hormonal balance and extending the implantation window, indicating its potential as a complementary therapy in ART (Hu et al.). Traditional Chinese medicine (TCM), specifically the Dingkun pill, was evaluated for its role in improving fertility outcomes in women with thin endometrium. When combined with conventional hormonal treatments, TCM significantly improved endometrial thickness, luteal function, and cumulative pregnancy rates, demonstrating the utility of integrating TCM in fertility management (Jin et al.).

## Sexual precocity and puberty suppression

In addressing sexual precocity, one notable study examined the combination of anastrozole and leuprorelin in treating early-maturing girls with compromised growth potential. The findings revealed that continuing anastrozole monotherapy after the combined treatment further improved near-adult height, offering a promising approach for managing growth outcomes in early puberty (Papadimitriou et al.). Puberty suppression (PS) in adolescents with gender dysphoria was another key focus. PS using GnRH analogs helps alleviate distress by halting the development of secondary sex characteristics, offering transgender adolescents more time to explore their gender identity. However, the long-term effects of PS, including bone health and reproductive potential, remain uncertain, necessitating further research (Betsi et al.).

## Advances in reproductive surgery and machine learning

A novel web-based nomogram developed using machine learning models helps predict the likelihood of spontaneous pregnancy following reproductive surgery. This tool incorporates clinical indicators such as age, AMH levels, and infertility duration, providing an individualized prediction of natural conception and helping couples make informed decisions about their fertility options (Liu et al.).

## Global trends and ethical considerations in ART

A bibliometric analysis of PCOS research over the past decade highlights trends in reproductive health, showing a growing focus on gut microbiota, microRNAs, and Vitamin D deficiency in PCOS management. This analysis provides insight into current research directions and identifies areas for future study, particularly in understanding the pathogenesis and treatment of PCOS (Shi et al.). In terms of ethical considerations, a survey of IVF add-ons in Japan revealed that many clinics offer treatments with limited supporting evidence. The study called for better regulatory oversight and patient counseling to ensure that ART patients receive evidence-based care, particularly when it comes to costly add-on treatments (Shionoya et al.). Another study addressing septate uterus correction through septum resection versus expectant management found no significant improvement in reproductive outcomes following septum resection. These results challenge the routine use of septum resection in patients with septate uterus, suggesting that a more cautious approach may be warranted (Liu et al.).

## Conclusion

This extensive body of research on sexual precocity and infertility demonstrates significant progress in reproductive health. Innovations in ART protocols, personalized hormone therapies, and complementary treatments such as acupuncture and traditional medicine have shown promising results. However, challenges remain, particularly in optimizing treatments for specific subgroups such as those with poor ovarian response, high BMI, or advanced male age. Furthermore, ethical concerns regarding the use of unproven ART add-ons and the long-term safety of puberty suppression highlight the need for continued research and regulatory oversight. By embracing both modern and traditional approaches, as well as integrating technological advancements such as machine learning, the future of reproductive medicine holds great promise. Continued exploration into the genetic and hormonal mechanisms underlying reproductive disorders will pave the way for more effective, individualized therapies and improved clinical outcomes.
